# 

**Published:** 1894-05

**Authors:** 


### THE AMERICAN JOURNAL OF DENTAL SCIENCE,

EDITED BY

F. J. S. GORGAS, M. D., D. D. S.

RICHARD GRADY, M. D., D. D. S.

VOLUME XXVIII—Third Series.

(1894-5.)

BALTIMORE:
SNOWDEN & COWMAN MFG. CO.,

LONDON:
TRUBNER & CO., 60 PATERNOSTER ROW.

1895.

SURGEON GENERAL'S OFFICE
152149.
LIBRARY.

### Index to Vol. XXVIII.—Third Series.

A New Practical Method of Fusing Platinum................ 1
Antiseptic Dentistry........................................ 8
An Act to Amend and Re-enact Sections 1767 and 1774 of the Code of Virginia............................................ 58
Admission to Study Dentistry............................... 65
American Dental Association................................ 86
A Praiseworthy Effort: A Reference Library for Dentists, 87
Aluminum Plates ............................................ 88
An Important Decision—The Law Regulating Dentistry in Tennessee Tested........................................ 89
American Dentistry in London............................... 90
A New Porcelain ............................................ 96
Anæsthesia ................................................. 142
A Dental Revolt............................................. 142
American Dentists .......................................... 144
A Suggestion upon the Preparation of the Fingers and Nails for Surgical Operations.................................. 156
Alloy for Gold Solders ..................................... 192
American Dental Society of Europe.......................... 233
A Case in Practice.......................................... 234
Amblyopia Caused by Dental Irritation...................... 236
An Original Crown........................................... 286
A Year's Experience with Three New Remedies................ 321
Aristol .................................................... 336
Artificial Crowns .......................................... 359
A Nice Appearing Lady Dentist.............................. 378
Alcohol in Shock............................................ 382
Abrasions of the Skin....................................... 419
A Cause for Baldness........................................ 422
A New Era in Dental Practice............................... 433
A Pathological Condition of the Mucous Glands of the Mouth...................................................... 459
An Ideal Business and Social Meeting of Dentists ......... 469
A Survival ................................................. 479
Alveolar Abscess ........................................... 509
Bacteriology................................................ 48
Bridge Work................................................. 71
Bleaching with Pyrozone..................................... 236
Blood Poisoning............................................. 288
Bicycle-Riding for Women.................................... 429

Bleaching Teeth........ 473
Bleachant........ 576
Combination of Cohesive and Non-Cohesive Gold in Filling. 79
Carborundum........ 140
Capping Dental Pulps........ 144
Correct Articulation in Bridge-work........ 192
Cocain Injection........ 192
Caries of the Alveolus........ 230
Cleaning the Teeth........ 236
Care of the Mouth in Sick Persons........ 247
Causes of Irregularities........ 328
Caries of the Alveolus........ 336
Comparison of Professional Fees........ 379
Calcification of Dental Pulps........ 400
Cold Water as an Antipyretic........ 424
Chloroform Nausea........ 428
Chloroform During Sleep........ 428
Coagulants........ 474
Coffee Kills Germs........ 475
Cast Solid Gold Cusps........ 528
Dentists of Maryland and Washington Meet........ 36
Death From Nitrous-Oxide Gas........ 62
Dental Dots........ 93
Decay of Teeth Coincident With Advanced Civilization........ 95
Death After Taking Gas........ 138
Discussion on the "Retention of Artificial Dentures in Edentulous Lower Jaws,"........ 176
Diagnosis of Pulp Stones........ 190
Dental Civilization........ 272
Dr. J. Austin Dunn's Syringe........ 285
Devitalizing Pulps........ 287
Dental Works in the Enoch Pratt Free Library........ 332
Discrimination in Filling Materials........ 337
Dental Department of Howard University........ 375
Disease of the Antrum........ 378
Died While Having a Tooth Extracted........ 381
Dental Legislation........ 385
Disinfection of Instruments........ 469
Dr. William H. Hoopes........ 470
Dry Surgery in Germany........ 477
Death is Painless........ 480
Dentists and the Census of 1890. Historical Sketch........ 481
Diagnosis in Obscure Cases........ 528
Delusive Pain........ 574
Electricity in 1900........ 45
Effects of Pregnancy........ 46

Electricity in Dentistry.... 188
Extracting for Irregularity.... 285
Erosion of the Teeth.... 408
Filling Children's Teeth.... 369
Free Artificial Teeth for Soldiers.... 375
Food and the Teeth.... 430
Filling Cervical Cavities.... 451
Failures to be Avoided.... 470
Filling Roots.... 471
Fast Living.... 478
Facial Neuralgia, from a Dento-Surgical Standpoint,.... 495
Filling Children's Teeth.... 518
Fractures of the Maxilla.... 551
Gum Lancing in Difficult Primary Dentition.... 120
Governor Brown Turned Down the Dentists' Bill.... 138
Gold in Pauper's Teeth Saved.... 239
How an Old Story Originated.... 44
Health and Happiness.... 47
How to Get Clean Joints.... 94
How to Polish Porcelain Teeth.... 141
Hypnotism.... 158
Hints Worth Remembering.... 191
Hot Water for Hemorrhage.... 235
Homœopathic Treatment of Toothache from Pulp-capping . 312
Hints.... 377
Hydrogen Dioxide.... 382
How Metals Get Tired.... 420
Hydrotherapy in Fractures.... 426
Hydrogen Dioxide as a Blood-Test.... 576
Illinois State Dental Society.... 35
Immediate Root Filling.... 97
Ideal Bridge-Work.... 345
Killing an Abscess.... 89
Keeping the Gingival Portion of a Cavity Dry.... 238
Love and Law.... 41
Lauder Brunton on Headaches.... 138
Local Obtundents.... 193
Little Aids in Practice.... 375
Manipulative Ability.... 7
Mr. Tomes' Inaugural Address.... 145
Mouth Poultice.... 288
Malpractice.... 362
Medical Antisepsis.... 424
Making Steel Crown Dies.... 473
Mineral Cements.... 547
Magnesium Hydrate.... 574

North-western University Dental School.................... 85
Nitrate of Silver as a Therapeutic Agent.................... 153
National Association of Dental Faculties.......... ....... 216
National Association of Dental Examiners.................. 294
Necrosis of the Alveolar Process: How shall it be Treated? 316
Nitrate of Silver and its Application in Diseased Conditions of the Mouth and Teeth.................. 325
Necrosis, Caused by the Non-eruption of a Deciduous Molar, 334
New Means of Local Anæsthesia.................. 425
Objection to Amalgam.................. 69
Opinion Delivered by President Judge Hanna, Orphans' Court, Philadelphia, in the Waesch's Estate Case......... 138
Oxiphosphate....... .................. 143
Observe, Digest, Concentrate, Act...... .. .................. 280
Our Present Status.................. 342
Putrescent Pulps.............. .................. 47
Pyorrhœa Alveolaris.... .................. 55
Pericementitis Producing Abscess.................. 73
Prof. R. B. Winder.................. 186
Protection of Cement Fillings.................. 191
Protrusion of the Upper Jaw.................. 249
Pulpitis.................. 288
Promiscuous Extraction of Teeth.................. 335
Pulp Canal Filling .................. 415
Porcelain Work........ .................. 453
Painless Extraction Thirty Years Ago .................. 478
Pulp-Treatment—Cap and Dressing.................. 526
Pulp Capping in Deciduous Teeth.................. 528
Porcelain Dental Art.................. 554
Queer Facts About Teeth.................. 116
Retention of Artificial Dentures.................. 25
Right-Sightedness and Left-Sightedness.................. 213
Remarks on the Treatment of Exposed Pulps and Pulpless Teeth.................. 289
Root Filling.................. 423
Saving the Teeth.................. 4
Storage Batteries and Their Uses in Dentistry.................. 19
Spongy Gums.................. 43
Some Remarks on the Treatment of Inflamed Pulp.......... 49
Southern Dental Association.................. 86
Swallowed Plates.................. 87
Soldering Aluminum.................. 141
Silver and Aluminum.................. 191
Sodium Peroxid Solution.................. 229
Some Notes on the Etiology of Pyorrhœa Alveolaris........ 244
Some Points on Bridgework.................. 270

Science in Dentistry.... 299
Some Remarks on the Root-canal Question.... 404
Sawdust Bread.... 474
Sensitive Dentine.... 528
Sulfuric Acid For Opening Root-Canals.... 564
The Physiology of Conception.... 29
The Cracking of Teeth in Soldering.... 33
The American Dental Society.... 35
The Medical Law of Maryland.... 37
Trichloracetic Acid.... 45
To Cut Off Crowns.... 48
Testing Gold and Silver.... 48
Treatment of Broken Impressions.... 96
The Cracking of Teeth in Soldering.... 106
To Properly Articulate a Set of Teeth.... 107
The Semi-Centennial of the Discovery of Anæsthesia.... 110
The Treatment of Pericementitis.... 111
Tin, and Its Combinations with Gold and Amalgam.... 130
The Fourth Molar.... 140
The Earliest Hospital on Record.... 143
The Immediate Root-Filling Craze.... 173
The Dental Meetings at Old Point, Va.... 185
The Dangers of Hypnotism.... 188
The Nails.... 189
To Set a Logan Crown.... 190
The Treatment of Typhoid Fever.... 223
The Use of Peroxide of Hydrogen as a Hæmostatic.... 237
To Secure Rubber Dam.... 240
Treatment of Epithelioma.... 240
The Matrix in Filling Teeth.... 241
Tin.... 264
The Southern Dental Association.... 276
The Value of Sugar and the Effect of Smoking on Muscular Work.... 281
To Band a Logan Crown.... 286
To Obtund Sensitive Dentine.... 287
The Present Status of Dental Knowledge in the Medical Profession.... 307
Tropa-cocain in Painless Extraction of Teeth.... 323
The First Quarterly Clinic of the Maryland State Dental Society.... 333
Treatment of Root Canals.... 335
The Treatment and Filling of Pulpless Teeth as Learned From Papers Read at the World's Dental Congress, A. D., 1893.... 350
The Contagion of the Communion Cup.... 380

The Effect of Exercise Upon the Teeth .................... 409
The Cause of Death from Chloroform........................ 421
Treatment of Apparent Death by Rhythmetical Traction Upon the Tongue.................................. 431
To Secure Perfect Articulation Between Natural and Substitute Teeth in Bridge-Work........................ 432
To Increase the Density and Hardness of Amalgam Fillings, 432
Tin and Amalgam Filling.............................. 472
Teething Powders..................................... 475
The Parts that do not Grow Old......................... 476
The Effects of Intense Cold Upon the Mind ............... 476
The Census of 1890.................................... 524
To Remove Plaster Impression from the Tray when it Hangs 527
The Immediate Treatment of the Uncovered Pulp........... 529
Tin and its Combinations with Gold and Amalgam.......... 538
The Census Bulletin................................... 567
The New Gas "Argon" and its Discovery.................. 568
The Tongue in Disease................................. 573
The Advent of Interruptions .......................... 575
To Stop a Hemorrhage from Tooth Extraction ............ 576
Uncommon Case of Salivary Calculus..................... 427
Utilizing Amalgam Waste............................... 571
Vulcanizing Rubber.................................... 545
What Dentists are Eligible to Membership in the American Medical Association............................ 86
What Dr. T. W. Brophy Says............................ 96
Why are We Weary...................................... 283
Why People Become Deaf................................ 427
What Hot Springs and Hot Baths Will Do................ 479
What Part Did Antitoxin Play?.......................... 575

### Communicated.

### THE AMERICAN DENTAL SOCIETY.

The American Dental Society of Europe will hold its nineteenth meeting at Geneva, Switzerland, Aug. 6th, 7th, and 8th, 1894. Last year this annual gathering was omitted, as many members wished to attend the Columbian Dental Congress. American colleagues who may be taking a summer-vacation abroad are cordially urged to combine with their plans for travel and sightseeing a visit to their professional brethren in Europe and to join in the various exercises of the occasion. Members of the profession everywhere will be welcome. Programmes may be had on application to the President, Dr. L. C. Bryan, of Basel, or to the undersigned,

CHAS. W. JENKINS, Secretary,
No. 1 Sonnenquai, Zurich.

---

### ILLINOIS STATE DENTAL SOCIETY.

At the thirtieth annual meeting of the Illinois State Dental Society, held at Springfield, May 8-11, 1894, the following officers were elected for the ensuing year:

J. W. Cormany, Mt. Carroll, President; S. F. Duncan, Joliet, Vice-President; Louis Ottofy, Chicago, Secretary; W. A. Stevens, Chicago, Treasurer; Grafton Munroe, Springfield Librarian.

The next meeting will be held at Galesburg, May 14-17, 1895.

LOUIS OTTOFY, Secretary,
Masonic Temple, Chicago.

### To Readers and Correspondents.

Communications intended for publication, and exchange journals and papers, should be addressed to the Editor of The American Journal of Dental Science, No. 845 N. Eutaw St. Baltimore, Md. All letters relating to the business of the Journal, or containing cash remittances, to be directed to Messrs. Snowden & Cowman, No. 9 W. Fayette Street, Baltimore, Md. Articles for publication should be received by the Editor at least two weeks previous to the first of the month on which the number in which it is designed for them to appear, is issued.

### CONTENTS:

Article I.—A New Practical Method of Fusing Platinum. By L E. Custer, B. S., D. D. S. - - - - - 1

" II.—Saving the Teeth. By W. Irving Thayer, D. D. S., M. D. - - - - - - - - 4

" III.—Manipulative Ability. - - - - - - - 7

" IV.—Antiseptic Dentistry. By Garrett Newkirk, M. D. - 8

" V.—Storage Batteries and Their Uses in Dentistry. By W. W. Vance, D. D. S. - - - - - 19

" VI.—Retention of Artificial Dentures. - - - - 25

" VII.—The Physiology of Conception. By Dr. A. D. Barr. 29

" VIII.—The Cracking of Teeth in Soldering. By L. P. Haskell, D. D. S. - - - - - - - 33

### COMMUNICATED.

The American Dental Society. - - - - - - - 35

Illinois State Dental Society. - - - - - - - 35

### EDITORIAL, Etc.

Dentists of Maryland and Washington Meet. - - - - - 36

The Medical Law of Maryland. - - - - - - - 37

Love and Law. - - - - - - - - - - 41

### MONTHLY SUMMARY.

Spongy Gums. - - - - - - - - - 43

How an Old Story Originated. - - - - - - - 44

Electricity in 1900. - - - - - - - - - 45

Trichloracetic Acid. - - - - - - - - - 45

Effects of Pregnancy. - - - - - - - - 46

Putrescent Pulps. - - - - - - - - - 47

Health and Happiness. - - - - - - - - 47

Bacteriology. - - - - - - - - - - 48

To Cut Off Crowns. - - - - - - - - - 48

Testing Gold and Silver. - - - - - - - - 48

☞ The American Journal of Dental Science will contain fifty pages of reading matter in each number, making a volume of about Six Hundred pages,

EXCLUSIVE OF ADVERTISEMENTS

